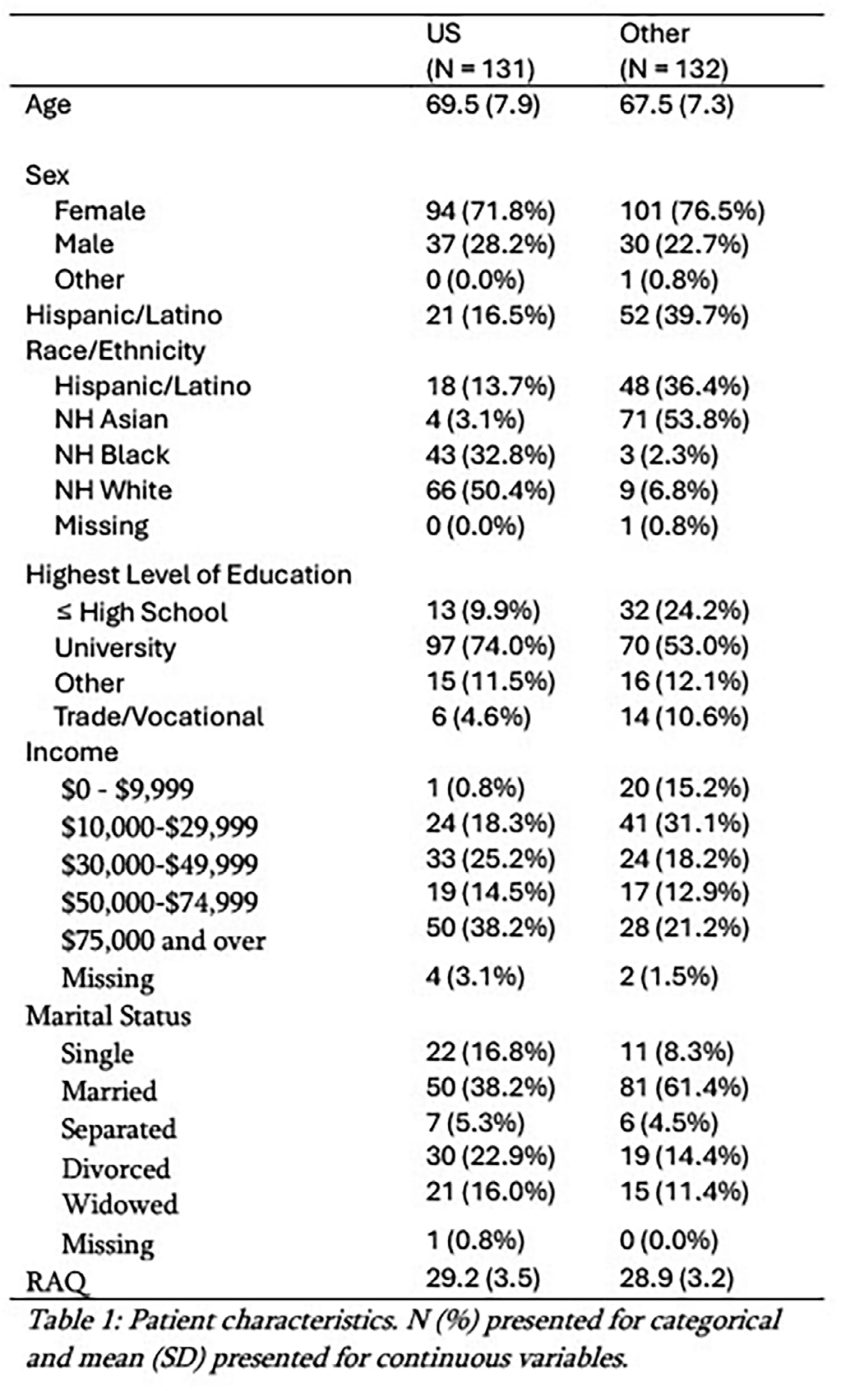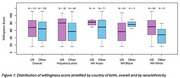# Nativity differences in willingness to participate in preclinical AD trials

**DOI:** 10.1002/alz70860_107722

**Published:** 2025-12-23

**Authors:** Edwin Duran, Michelle M Nuño, Megan G Witbracht, Kristin Harkins, Jason Karlawish, Christian R Salazar, Josh D Grill

**Affiliations:** ^1^ Institute for Memory Impairments and Neurological Disorders, University of California, Irvine, Irvine, CA, USA; ^2^ University of Southern California, Los Angeles, CA, USA; ^3^ University of Pennsylvania Perelman School of Medicine, Philadelphia, PA, USA; ^4^ The UC Irvine Institute for Memory Impairments and Neurological Disorders, Irvine, CA, USA

## Abstract

**Background:**

Several racial and ethnic populations are underrepresented in preclinical Alzheimer's disease (AD) trials. Research has shown that foreign‐born populations differ in terms of assimilation, education and socioeconomic status compared to US‐born participants, yet little research has explored whether and how such differences affect decisions to enroll in preclinical AD trials. We explored differences in willingness to learn AD biomarker status and enroll in preclinical AD trials between foreign‐ and US‐born participants.

**Method:**

We recruited a diverse sample of cognitively unimpaired older adults through community outreach. Mixed methods data were collected via cognitive interviews in English, Spanish, and Korean. Participants completed a conjoint experiment where they rated their willingness to participate in 16 trial scenarios using a 7‐point Likert scale, with total scores ranging from 16‐112. We collected self‐reported sociodemographic information, including country of origin and duration of time in the US. We will use linear regression and proportional odds models to quantify the association between nativity and willingness, summing responses across all trial vignettes, controlling for potential confounding variables including age, sex, and comorbidities.

**Result:**

Of the 263 participants who reported country of origin,132 (76.5% female) were foreign born and 131 (71.8% female) US born (Table 1). Foreign‐born participants were slightly younger (mean age = 67.5 (SD = 7.3) vs. 69.5 (SD = 7.9)) and more likely to be NH Asian and Hispanic/Latino (53.8% vs. 3.1% and 36.4% vs.13.7%) compared to US‐born participants. Figure 1 presents the distribution of total willingness scores stratified by country of birth and by race and ethnicity. US‐born participants had a mean willingness score of 63.47 (SD = 29.2, median = 68, IQR = 43‐87) and foreign‐born participants had a mean score of 60.3 (SD = 29.9, median = 64, IQR = 32‐82.5) (Figure 1).

**Conclusion:**

Recruitment strategies often overlook the sociocultural factors that shape the decision to participate in clinical trials for AD. The preliminary data collected can offer valuable insights for developing more efficient and inclusive approaches to recruitment and biomarker disclosure.